# Pelvic organ prolapse and uterine preservation: a cohort study (POP-UP study)

**DOI:** 10.1186/s12905-021-01208-5

**Published:** 2021-02-17

**Authors:** Daniel Gagyor, Vladimir Kalis, Martin Smazinka, Zdenek Rusavy, Radovan Pilka, Khaled M. Ismail

**Affiliations:** 1 Department of Obstetrics and Gynaecology, Faculty of Medicine and Dentistry, Palacky University and Faculty Hospital, Olomouc, Czech Republic; 2 Biomedical Center, Faculty of Medicine in Pilsen, Charles University, Pilsen, Czech Republic; 3 Department of Obstetrics and Gynecology, University Hospital, Pilsen, Czech Republic; 4 Department of Gynecology and Obstetrics, Faculty of Medicine in Pilsen, Charles University, Pilsen, Czech Republic

**Keywords:** Laparoscopic, Sacrocolpopexy, Cervicopexy, Hysteropexy, LSC, LSCH + LSC, TLH + LSC, LSH, POP-Q, Mesh, PFDI, PGI-I, Compartment

## Abstract

**Background:**

Abdominal and laparoscopic sacro-colpopexy (LSC) is considered the standard surgical option for the management of a symptomatic apical pelvic organ prolapse (POP). Women who have their uterus, and for whom an LSC is indicated, can have a laparoscopic sacro-hysteropexy (LSH), a laparoscopic supra-cervical hysterectomy and laparoscopic sacro-cervicopexy (LSCH + LSC) or a total laparoscopic hysterectomy and laparoscopic sacro-colpopexy (TLH + LSC). The main aim of this study was to compare clinical and patient reported outcomes of uterine sparing versus concomitant hysterectomy LSC procedures.

**Methods:**

A retrospective analysis of clinical, imaging and patient reported outcomes at baseline, 3 and 12 months after LSH versus either LSCH + LSC or TLH + LSC between January 2015 and January 2019 in a tertiary referral urogynecology center in Pilsen, the Czech Republic.

**Results:**

In total, 294 women were included in this analysis (LSH n = 43, LSCH + LSC n = 208 and TLH + LSC n = 43). There were no differences in the incidence of perioperative injuries and complications. There were no statistically significant differences between the concomitant hysterectomy and the uterine sparing groups in any of the operative, clinical or patient reported outcomes except for a significantly lower anterior compartment failure rate (*p* = 0.017) and higher optimal mesh placement rate at 12 months in women who had concomitant hysterectomy procedures (*p* = 0.006).

**Conclusion:**

LSH seems to be associated with higher incidence of anterior compartment failures and suboptimal mesh placement based on postoperative imaging techniques compared to LSC with concomitant hysterectomy.

**Supplementary Information:**

The online version contains supplementary material available at 10.1186/s12905-021-01208-5.

## Background

It is estimated that one in three women are affected by pelvic organ prolapse (POP) and one in 10 require a surgical procedure for its correction during their lifetime [1, 2]. POP is associated with numerous bothersome clinical symptoms including pelvic discomfort, vaginal bulge, urinary incontinence, urinary tract symptoms, fecal incontinence or sexual dysfunction. These often have a significant negative impact on their quality of life (QOL) or, even, serious life threatening consequences [3–8]. There is no significant correlation between the severity of clinical symptoms and the stage of POP, but there is a correlation between clinical symptoms and location of the underlying defect [9]. Anterior compartment prolapse tends to be associated with urgency symptoms requiring surgical intervention in the majority of cases [10], while posterior compartment prolapse is more likely to be associated with distal bowel dysfunction [11, 12].

Conservative management of apical prolapse is commonly used as first line treatment in general and the main option for women who have not completed their childbearing or those deemed to be at high operative risks. Nonetheless, surgical correction is an option that should always be discussed when counseling women about their treatment options. POP with a dominant apical defect can be treated using a number of surgical approaches and this choice can be one of the most challenging problems in urogynecology [13, 14]. However, high level evidence indicates that abdominal and laparoscopic sacro-colpopexy (LSC) result in better anatomical outcomes compared to sacrospinous ligament fixation and transvaginal mesh insertion [14]. Women who have their uterus and opt for a laparoscopic approach have several surgical options to consider; sacro-colpopexy, uterosacral ligament colpopexy [15], lateral ligament suspension or pecto-colpopexy [16]. Based on the currently available evidence, LSC is the most commonly used laparoscopic method and this could be in the form of laparoscopic sacro-hysteropexy (LSH), laparoscopic supra-cervical hysterectomy and laparoscopic sacro-cervicopexy (LSCH + LSC) or total laparoscopic hysterectomy and laparoscopic sacro-colpopexy (TLH + LSC).

The American College of Obstetricians and Gynecologists considers involving and supporting patients in the discussion about uterine preservation in elective surgery as obligatory [17]. Furthermore, there seems to be an increasing tendency for women to explore uterine preserving procedures for their POP surgical management rather than just accept a hysterectomy [18–20]. Therefore, increasing the availability of options, that do not necessitate a hysterectomy, gives women viable options to individualize their POP management plan. Nonetheless, one of the important determinants of women’s choice about uterine preservation or concomitant hysterectomy is the outcome associated with either procedure [18–20]. There is evidence that the route of concomitant hysterectomy during LSC does not seem to be associated with the perioperative or postoperative outcomes [21, 22]. However, at present, there is heterogenous information about comparative anatomical and functional outcomes with no comprehensive analysis based on whether the uterus was spared or removed [23–26]. Furthermore, there is paucity of information on surgical outcomes including mesh placement on postoperative imaging.

## Methods

The main aim of the study was to compare the clinical and patient reported outcomes of uterine sparing versus concomitant hysterectomy LSC procedures for a symptomatic apical POP. As a secondary aim we wanted to assess the peri- and postoperative complications associated with these procedures as an indicator of their safety profile.

This is a retrospective cohort study undertaken in a tertiary referral urogynecology center in Pilsen, the Czech Republic. All women referred with an intact uterus and a symptomatic apical POP and who were listed for one of the LSC procedures between January 2015 and January 2019 were included in our analysis. For the purpose of this study, we were interested in comparing women who had an LSH (uterine preservation) versus LSCH + LSC or TLH + LSC (concomitant hysterectomy). Local ethics committee approval was granted for the study. All patients included in this study provided written informed consent for the procedure and for the future use of their perioperative and follow-up data. The departmental medical database was used to gather data on patients’ demographics, medical history, history of abdominal and/or gynecological surgery, previous reconstructive POP surgery, obstetric history, urinary or bowel symptoms and POP-Q staging points [27, 28]. We also collected data on hospital length of stay (LOS). Extended LOS was defined as hospitalization longer than the 75th percentile [29]. The impact of the woman’s symptoms on her quality of life during the pre- and postoperative periods was assessed using the Pelvic Floor Distress Inventory (PFDI). This is a validated quality-of-life questionnaire consisting of a Urinary Distress Inventory (UDI), Pelvic Organ Prolapse Distress Inventory (POPDI) and a Colorectal-Anal Distress Inventory (CRADI). UDI and POPDI have a score range of 0 (least impact) to 300 (greatest adverse impact) while CRADI has a range of 0 to 400 and an overall summary PFDI score ranging from 0 to 1000 [30]. Perioperative complications were categorized according to the Dindo-Clavien classification [31].

Surgical procedures were performed by one of four experienced urogynecological subspecialists. We used the same surgical technique, sutures and mesh materials for all LSC variants as previously published by our group [32, 33].

In the research unit, postoperative follow-up appointments are routinely arranged at 3 and 12 months for assessment of the impact of surgery on the woman’s clinical symptoms, evaluation of any postoperative complications and clinical examination including a POP-Q measurement. In addition to the PFDI, their overall satisfaction with the surgical procedure is routinely evaluated by means of a 7-point Patient Global Impression of Improvement (PGI-I) scale ranging from "Very much worse” (PGI-I = 7) to "Very much better” (PGI-I = 1) [34]. Any identified mesh related complications are reported using the current standardized international classification [35]. A 3D/4D transperineal ultrasound scans is also routinely performed at both follow-up appointments to assess the bladder neck and mesh positions. The ultrasound protocol has been previously published and is derived from the standardized assessment protocol suggested by Dietz et al. [32, 33, 36]. Optimal mesh placement is assessed based on a set of composite parameters including: distance of the lowest margin of the anterior mesh strand from the bladder neck < 20 mm [32, 33]; shape of the mesh; absence of folding; and a vertical mesh descent on Valsalva ≤ 20 mm.

For the purpose of this study, anatomical apical compartment failure was defined as a postoperative POP-Q point C ≥ -TVL/2 cm (apical descent lower than half of the vaginal length). Points Ba and Bp ≥ − 1 cm were considered failure in the anterior and posterior compartment respectively. Subjective success of the procedure was defined as a PGI-I < 3 i.e. "Very Much" or "Much Better". Statistical analysis was performed using IBM SPSS Statistics software version 22 (Armonk, NY: IBM Corp.). A p < 0.05 was considered statistically significant.

In addition to the comparisons between uterine sparing versus concomitant hysterectomy LSC procedures, we undertook a sub-analysis comparing the three procedures (LSC, LSCH + LSC and TLH + LSC) to each other.

## Results

**Fig. 1:**
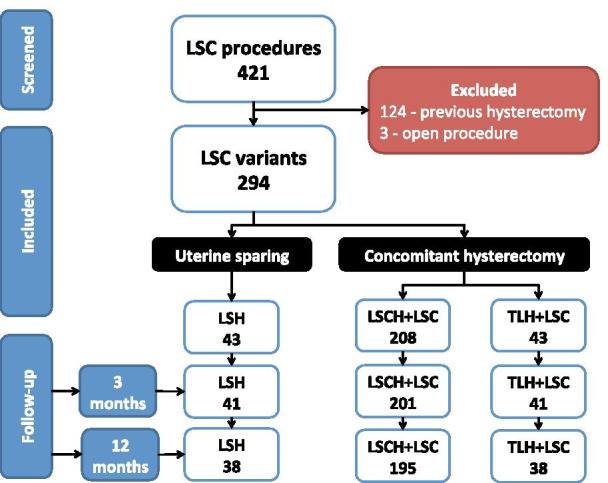
Flowchart of study participants

A total of 421 LSC procedures were performed during the study period. Of these, 124 (29.5%) procedures were performed on women who previously had a hysterectomy and hence excluded from this study. A further 3 patients (0.7%) were not included because they had their procedure performed through a laparotomy. The remaining 294 (70.0%) women who have had one of the LSC variants for apical POP management were all included in our analysis. These included 43 (14.6%) women who had a uterine sparing procedure LSH and 251 (85.4%) who had a concomitant hysterectomy, where 208 (70.8%) had LSCH + LSC and 43 women (14.6%) had a TLH + LSC (Fig. 1).

Table 1 and Additional file 1 summarize participants’ characteristics, preoperative POP-Q and PFDI scores grouped by whether the uterus was preserved or not and by type of procedure respectively. There were significant differences between the cohort of women who had LSH compared to LSCH + LSC/TLH + LSC with regards to BMI (25.2 kg/m^2^ vs. 26.6 kg/m^2^, *p* = 0.006), age (49.0 years vs. 64.0 years, *p* < 0.0001) and comorbidities like cardiovascular disease (20.9% vs. 55.8%, *p* < 0.0001) and diabetes (2.3% vs. 15.5%, *p* = 0.02). There was also a significant difference in POP-Q staging based on point Ba between the 2 groups (*p* < 0.0001) (Table 1). There were significant differences in reported urinary hesitancy (30.2% vs. 49.0%, *p* = 0.023) and constipation (9.3% vs. 23.1%, *p* = 0.04) between both cohorts. However, no significant differences were found in other pre-operative POP-Q parameters, reported urinary or anal incontinence, or preoperative PFDI score.

**Table 1:** Demographic details of study cohorts

Variable		Total population N = 294	Uterine sparing (LSH) N = 43	Concomitant Hysterectomy (LSCH + LSC & TLH + LSC) N = 251	*p*
BMI [Median (range)]		26.4 (17.7–37.2)	25.2 (17.7–31.6)	26.6 (19.2–37.2)	0.006^a^
Age [Median (range)]		63.0 (28–84)	49.0 (28–70)	64.0 (37–84)	< 0.0001^a^
Parity [Median (range)]		2.0 (0–9)	2.0 (1–5)	2.0 (0–9)	0.063^a^
Cardiovascular disease [N (%)]		149 (50.7%)	9 (20.9%)	140 (55.8%)	< 0.0001^b^
Diabetes mellitus [N (%)]		40 (13.6%)	1 (2.3%)	39 (15.5%)	0.020^b^
Previous DVT or pulmonary embolism [N (%)]		39 (13.3%)	3 (7.0%)	36 (14.3%)	0.188^b^
Asthma [N (%)]		22 (7.5%)	1 (2.3%)	21 (8.4%)	0.219^c^
Previous abdominal surgical history [N (%)]		137 (46.6%)	17 (39.5%)	120 (47.8%)	0.315^b^
Previous gynecologic surgery [N (%)]		90 (30.6%)	15 (34.9%)	85 (33.9%)	0.896^b^
Previous POP surgery [N (%)]		6 (2.0%)	2 (4.7%)	4 (1.6%)	0.214^c^
Point C	POP Q stage I	19 (6.5%)	5 (11.6%)	14 (5.6%)	0.073^b^
	POP Q stage II	159 (54.1%)	24 (55.8%)	135 (53.8%)	
	POP Q stage III	78 (26.5%)	11 (25.6%)	67 (26.7%)	
	POP Q stage IV	38 (12.9%)	3 (7.0%)	55 (21.9%)	
Point Ba	POP Q stage I	3 (1.0%)	1 (2.3%)	2 (0.8%)	< 0.0001^b^
	POP Q stage II	65 (22.1%)	28 (65.1%)	37 (14.7%)	
	POP Q stage III	165 (56.1%)	11 (25.6%)	154 (61.4%)	
	POP Q stage IV	51 (17.4%)	3 (7.0%)	48 (19.1%)	
Point Bp	POP Q stage I	96 (32.7%)	13 (30.2%)	83 (33.1%)	0.634^b^
	POP Q stage II	132 (44.9%)	22 (51.2%)	110 (43.8%)	
	POP Q stage III	46 (15.6%)	7 (16.3%)	39 (15.5%)	
	POP Q stage IV	20 (6.8%)	1 (2.3%)	19 (7.6%)	
Stress urinary incontinence [N (%)]		87 (29.6%)	11 (25.6%)	76 (60.3%)	0.533^b^
Urge urinary incontinence [N (%)]		66 (22.4%)	8 (18.6%)	58 (23.1%)	0.513^b^
Hesitancy: a delay in initiating micturition [N (%)]		136 (46.3%)	13 (30.2%)	123 (49.0%)	0.023^b^
Urinary retention [N (%)]		126 (42.9%)	21 (48.8%)	115 (45.8%)	0.714^b^
Constipation [N (%)]		62 (21.1%)	4 (9.3%)	58 (23.1%)	0.040^b^
Anal incontinence [N (%)]		102/266 (38.3%)	16/41 (39.0%)	86/225 (38.2%)	0.923^b^
Pre-op UDI [median (range)]		51.2 (0–189)	52.6 (5.8–164)	51.2 (0–189)	0.481^a^
Pre-op POPDI [median (range)]		68.5 (0–282)	58.9 (10.7–152)	69.6 (0–282)	0.204^a^
Pre-op CRADI [median (range)]		35.1 (0–216)	34.2 (0–164)	36.4 (0–216)	0.963^a^
Pre-op PFDI [median (range)]		171.7 (0–600)	148.0 (16.5–442)	1712.4 (0–600)	0.524^a^

^a^ Mann–Whitney U test; ^b^ Chi-square Test; ^c^ Fisher’s exact Test

BMI: body mass index, DVT: deep venous thromboembolism

Operative characteristics and postoperative complications are presented in Table 2 and Additional file 2. Based on the Dindo-Clavien classification, there were no differences in the incidence of perioperative injuries and complications. However, operating time and blood loss were higher in the concomitant hysterectomy compared to the uterine sparing group (125 min vs. 120 min, *p* = 0.052).

**Table 2:** Peri-operative characteristics amongst study cohorts

Variable	Total population N = 294	Uterine sparing (LSH) N = 43	Concomitant Hysterectomy (LSCH + LSC & TLH + LSC) N = 251	*p*
Operating time [min] [Median (range)]	120.5 (60–240)	120.0 (70–225)	125.0 (60–240)	0.052^a^
Operating time more than 3 h [N (%)]	16 (5.4%)	2 (4.7%)	14 (5.6%)	1.000^b^
Blood loss [ml] [Median (range)]	150 (50–1400)	150 (50–1400)	200 (50–800)	0.259^a^
Estimated blood loss more than 300 ml [N (%)]	14 (4.7%)	2 (4.7%)	12 (4.8%)	1.000^b^
Perioperative blood transfusion	2 (0.7%)	1 (2.3%)	1 (0.4%)	0.286^b^
Bladder injury [N (%)]	10 (3.4%)	2 (4.7%)	8 (3.2%)	0.657^b^
Rectal injury [N (%)]	0 (0.0%)	0 (0.0%)	0 (0.0%)	–
Vaginal injury [N (%)]	2 (0.7%)	0 (0.0%)	2 (0.8%)	1.000^b^
Early postoperative complications Dindo-Clavien grade 0 [N (%)]	281 (95.6%)	41 (95.3%)	240 (95.6%)	0.566^b^
Early postoperative complications Dindo-Clavien grade I [N (%)]	6 (2.0%)	1 (2.3%)	5 (2.0%)	
Early postoperative complications Dindo-Clavien grade II [N (%)]	3 (1.0%)	1 (2.3%)	2 (0.8%)	
Early postoperative complications Dindo-Clavien grade III [N (%)]	4 (1.4%)	0 (0.0%)	4 (1.6%)	
Prolonged hospitalization [N (%)]	6 (2.0%)	2 (4.7%)	4 (1.6%)	0.234^b^

^a^ Mann–Whitney U test; ^b^ Fisher’s Exact Test

Table 3 shows anatomical and functional outcomes at 3 and 12 months postoperative. When comparing outcomes in women who had a concomitant hysterectomy at the time of LSC compared to LSH, there were no statistically significant differences in any of the clinical or patient reported outcomes except for a significantly higher anterior compartment failure rate at 12 month follow-up as assessed by POP-Q in women who had a uterine sparing procedure (21.1% vs. 7.7%, *p* = 0.017) (Table 3, Additional file 3).

**Table 3:** Post-operative follow-up at 3 months and at 12 months

	3 month follow-up N = 283				12 month follow-up N = 271			
	Total N = 283	Uterine sparing (LSH) N = 41	Concomitant hysterectomy (LSCH + LSC & TLH + LSC) N = 242	p	Total N = 271	Uterine sparing (LSH) N = 38	Concomitant hysterectomy (LSCH + LSC & TLH + LSC) N = 233	*p*
Postoperative mesh complications [N/N] (%)	2/283 (0.7%)	0/41 (0.0%)	2/242 (0.8%)	1.000^b^	4/271 (1.5%)	1/38 (2.6%)	3/233 (1.3%)	0.456^b^
Failure in apical compartment Point C ≥ -TVL/2 [N/N] (%)	0/283 (0.0%)	0/41 (0.0%)	0/242 (0%)	–	0/271 (0.0%)	0/38 (0.0%)	0/233 (0%)	–
Failure in anterior compartment Point Ba ≥ 1 [N/N] (%)	12/283 (4.2%)	4/41 (9.8%)	8/242 (3.3%)	0.079^b^	26/271 (9.6%)	8/38 (21.1%)	18/233 (7.7%)	0.017^b^
Failure in posterior compartment Point Bp ≥ -1 [N/N] (%)	14/283 (4.9%)	0/41 (0.0%)	14/242 (5.8%)	0.114^b^	15/271 (5.5%)	0/38 (0.0%)	15/233 (6.4%)	0.140^b^
PGI-I 1, 2 [N/N] (%)	243/283 (85.9%)	35/41 (85.4%)	208/242 (86.0%)	0.607^b^	255/271 (94.1%)	33/38 (86.8%)	222/233 (95.3%)	0.055^b^
PGI-I 3 [N/N] (%)	28/283 (9.9%)	4/41 (9.8%)	24/242 (9.9%)		11/271 (4.0%)	3/38 (7.9%)	8/233 (3.4%)	
PGI-I 4 [N/N] (%)	8/283 (2.8%)	1/41 (2.4%)	7/242 (2.9%)		4/271 (1.5%)	2/38 (5.3%)	2/233 (0.9%)	
PGI-I 5 [N/N] (%)	2/283 (0.7%)	1/41 (2.4%)	1/242 (0.4%)		1/271 (0.4%)	0/38 (0.0%)	1/233 (0.4%)	
PGI-I 6 [N/N] (%)	2/283 (0.7%)	0/41 (0.0%)	2/242 (0.8%)		0/271 (0.0%)	0/38 (0.0%)	0/233 (0%)	
PGI-I 7 [N/N] (%)	0/286 (0.0%)	0/41 (0.0%)	0/242 (0%)		0/271 (0.0%)	0/38 (0.0%)	0/233 (0%)	
Δ UDI pre-op – post-op [median (range)]	20.1 (− 159 to 153)	17.9 (− 54.9 to 131)	20.1 (− 159 to 153)	0.988^a^	25.0 (− 112 to 160)	17.6 (− 99 to 160)	33.7 (− 112 to 150)	0.585^a^
Δ POPDI pre-op – post-op [median (range)]	40.5 (− 112 to 256)	35.7 (− 56 to 127)	41.1 (− 112 to 256)	0.559^a^	39.3 (− 74 to 253)	30.4 (− 43 to 135)	48.2 (− 189 to 253)	0.502^a^
Δ CRADI pre-op – post-op [median (range)]	7.7 (− 189 to 199)	10.0 (− 41 to 129)	7.1 (− 189 to 199)	0.338^a^	3.6 (− 92 to 170)	10.7 (− 38 to 112)	7.1 (− 118 to 170)	0.187^a^
Δ PFDI pre-op – post-op [median (range)]	46.1 (− 342 to 450)	50.7 (− 206 to 373)	59.5 (− 343 to 450)	0.889^a^	70.4 (− 182 to 460)	66.9 (− 123 to 281)	82.5 (− 338 to 460)	0.960^a^

^a^ Mann–Whitney U test; ^b^ Fisher’s Exact Test; UUI: Urge urinary incontinence; SUI: Stress urinary incontinence

Moreover, concomitant hysterectomy procedures were likely to be associated with absent mesh folding on at 3 (94.7% vs. 80.0%, p = 0.004) and 12 months (93.8% vs. 82.1%, p = 0.021) and optimal composite mesh placement at 12 months (81.7% vs. 67.6%, *p* = 0.006) as assessed by ultrasonography (Table 4).

**Table 4:** Mesh placement on transperineal scanning at 3 months and at 12 months

	3 month follow-up N = 283				12 month follow-up N = 271			
	Total N = 283	Uterine sparing (LSH) N = 41	Concomitant hysterectomy (LSCH + LSC & TLH + LSC) N = 242	p	Total N = 271	Uterine sparing (LSH) N = 38	Concomitant hysterectomy (LSCH + LSC & TLH + LSC) N = 233	*p*
Regular shape of the mesh upon visualization of the whole mesh [N/N](%)	244/266 (91.7%)	35/40 (87.5%)	209/226 (92.5%)	0.345^b^	238/265 (89.8%)	32/39 (82.1%)	206/226 (91.2%)	0.090^b^
No folding of the mesh [N/N](%)	248/268 (92.5%)	32/40 (80.0%)	216/228 (94.7%)	0.004^b^	245/266 (92.1%)	32/39 (82.1%)	213/227 (93.8%)	0.021^b^
No mesh descent on Valsalva 196/226 (86.7%) [N/N](%)	266/268 (99.3%)	39/40 (97.5%)	227/228 (99.6%)	0.277^b^	252/254 (99.2%)	36/37 (97.3%)	216/217 (99.5%)	0.271^b^
Overall evaluation: all criteria for a properly placed mesh fulfilled [N/N](%)	227/266 (85.3%)	31/40 (77.5%)	196/226 (86.7%)	0.146^b^	214/254 (84.3%)	25/37 (67.6%)	189/217 (81.7%)	0.006^b^

^a^ Mann–Whitney U test; ^b^ Fisher’s Exact Test

On subgroup analysis, the only significant difference was that the operative time was longer in the TLH + LSC subgroup compared to the LSH subgroups (140 min vs. 120 min, *p* = 0.048). Furthermore, blood loss was significantly higher when comparing TLH + LSC to LSH (250 ml vs. 150 ml, *p* = 0.001) and TLH + LSC to LSCH + LSC (250 ml vs. 150 ml, *p* < 0.0001).

## Discussion

### Summary of findings

This is among the first studies comparing outcomes of the different variants of LSC with a particular focus on comparing these outcomes based on whether the uterus was spared or concomitantly removed. Of the total number of women who had an LSC procedure during the study period, 70% of women who presented with a significant apical POP requiring surgery had their uterus in situ. The majority of these women had a concomitant hysterectomy at the time of LSC. Our study demonstrated that LSC procedures with a concomitant total hysterectomy were associated with statistically significantly longer operating time and intra-operative blood loss. However, the median differences between groups were only 5 min and 50 ml respectively. In contrast, uterine sparing LSCs were associated with a significantly higher likelihood of a suboptimally placed mesh at 3 and 12 months postoperative and anterior compartment failures at 12 months. Nevertheless, other anatomical and patient reported outcomes were comparable in both groups. On head to head comparison of the different LSC variants there was no significant difference in anterior compartment failure rates. However, this observation should be interpreted with caution due to the small samples in some of the subgroups.

### Results in relation to what is known

Other groups have reported higher incidence of anatomical failures in association with LSH [23, 24]. Saliba et al. compared outcomes of 64 LSCH + LSC versus 12 LSH procedures and the anatomical failure, defined as POP stage ≥ 2, was significantly higher in the LSH groups in both any and apical compartments (33.3% vs. 6.2% and 16.7% vs. 0% respectively), however, the study authors did not provide the actual length of follow-up [24]. Similarly, Gracia and colleagues reported significantly higher apical compartment failures, defined as C stage ≥ 2, when comparing 12 months outcomes after 15 LSH compared to 30 LSCH + LSC (53.2% vs. 10.0%). Anterior compartment recurrence (Ba stage ≥ 2) was also more common in their LSH cohort (72.4% vs. 33.3%) [23]. The reported incidence of anterior compartment failures concur with our findings of 21.1% vs. 8.8% in our LSH and LSCH + LSC subgroups respectively. Nevertheless, our low incidence of apical compartment recurrences both in the main and subgroup analyses are in stark contrast to the rates reported in these studies.

When comparing LSH and TLH + LSC, we did not have any apical compartment recurrences at 12 months compared to Pan et al. who reported 13.9% and 5.9% recurrence rates for the equivalent procedures in a cohort of 65 and 34 women who had LSH and TLH + LSC respectively, albeit, after an average follow-up of 34 months. Moreover, their anterior compartment failure rates were 13.9% versus 11.8% compared to 21.1% versus 5.2% in our study, while their posterior compartment recurrence incidences were 4.6% versus 5.9% and it was 0% and 15.8% in our LSH and TLH + LSC groups respectively [25]. The identified posterior compartment failure rate in our TLH + LSC was also higher than that reported by Illiano and associates (15.8% compared to 2.4%) [26]. Due to the nature of our study we were not able to explore the reasons behind the aforementioned differences in recurrence rates between our study and previous reports, which could be related to the operative technique, patient selection or duration of follow-up. Another reason for discrepancy in reported outcome rates between various studies is the POP-Q cut-off used to determine failure. Indeed, if we use the Ba > 0 cut-off for cystocele recurrence adopted in other studies [32, 37]**,** our anterior compartment failure rates would have dropped to zero.

We identified a significantly higher likelihood of suboptimal mesh placement in our LSH group, which probably is an indicator of the relative technical difficulty of inserting the mesh in LSH compared to other variants of LSC. It would be of interest to assess if there is any association between mesh placement and clinical outcomes. However, we did not undertake such analysis because we believe our study is underpowered to test such hypothesis. The incidence of postoperative mesh-related complications in our study falls within the range of 1.0–2.6%. However, the incidence of mesh erosions were similar in our subgroup analyses unlike the differences reported by other authors [38–40]. It is the technical challenge to achieve proper placement of the anterior mesh in LSH and be able to create a “de novo vaginal apex” that is considered to be a plausible reason for the higher anterior compartment failure in association with LSH and is the driver behind the suggestion of alternative modifications to the standard technique [41]. The process of refining the current LSH technique is crucially important since the number of women opting for uterine sparing surgery significantly falls if this technique is associated with inferior anatomical outcome [18, 19].

### Strengths and limitations

We appreciate that there are some limitations to our work. First, the retrospective nature of the study has an inherent risk of introducing selection and recall bias into our data. Due to the rigor in our hospital database and the high level of specialism required for the surgical procedures being assessed, it is extremely unlikely we would have missed any procedures or data that was collected. However, the issue of selection bias is more challenging to tackle except within a context of a randomized trial. Indeed, our 2 groups of interest had significant differences in their demographics and associated comorbidities. Second, although we report 12-month follow-up data, in POP surgery, this is considered relatively short. We recognize that the longer the follow-up the higher attrition rate, hence, the current study will form the basis for our LSC database that will enable us to increase our sample size and assess longer term outcomes. Although our sample size in the uterine sparing cohort was relatively small, a post hoc power calculation showed that the power of our study to identify the difference in anterior compartment failure rates between our main cohorts at a significance level of 0.05 was 70% (Additional file 4). Finally, it could be perceived that a report from a single center might limit the external validity of the study. However, the involvement of several independent trained surgeons, in a center accredited by the European Board & College of Obstetrics and Gynaecology (EBCOG) for training and the use of standardized operative technique and validated outcome measures make our findings generalizable. In contrast, the reporting on LSC variants based on whether the uterus was removed or spared using a comprehensive set of core outcomes and the novelty of the postoperative imaging information are major strengths to our study.

## Conclusion

Many women referred with a symptomatic apical POP have their uterus in situ. LSH was associated with higher incidence of anterior compartment failures and suboptimal mesh placement compared to LSC with concomitant hysterectomy. LSCH + LSC appears to have the best balance between limiting operative time and blood loss against recurrence rates at 12 months. The availability of longer-term outcomes for the different LSC variants and the assessment of proposed new modifications to overcome challenges to mesh placement in LSH are essential to give women realistic prospects of making an equitable informed choice.

## Supplementary Information

**Additional file 1**. Demographic data amongst women undergoing LSH, LSCH+LSC and TLH+LSC

**Additional file 2**. Peri-Operative characteristics amongst women undergoing LSH, LSCH+LSC and TLH+LSC

**Additional file 3**. Post-operative follow-up at 3 months and at 12 months.

**Additional file 4**. Post hoc power calculation

## Data Availability

The datasets used and/or analysed during the current study are available from the corresponding author on reasonable request.

## Abbreviations

CRADI: Colorectal-Anal Distress Inventory
LSC: Laparoscopic sacro-colpopexy
LSCH + LSC: Laparoscopic supra-cervical hysterectomy and Laparoscopic sacro-cervicopexy
LSH: Laparoscopic sacro-hysteropexy
PGI-I: Patient Global Impression of Improvement
POP: Pelvic organ prolapse
POPDI: Pelvic Organ Prolapse Distress Inventory
POP-Q: Pelvic organ prolapse quantitation system
PFDI: Pelvic Floor Distress Inventory
TLH + LSC: Total laparoscopic hysterectomy and laparoscopic sacro-colpopexy
QOL: Quality of life
UDI: Urinary Distress Inventory
