# Supplementary material for: Pelvic organ prolapse and uterine preservation: a cohort study (POP-UP study)
Source: BMC Womens Health. 2021 Feb 17;21:72. doi: 10.1186/s12905-021-01208-5 (PMC7890869; doi:10.1186/s12905-021-01208-5)
Supplement: Supplementary file 1 — Additional file 1. Demographic data amongst women undergoing LSH, LSCH+LSC and TLH+LSC [file 12905_2021_1208_MOESM1_ESM.docx]

**Additional file 1: Demographic data amongst women undergoing LSH, LSCH+LSC and TLH+LSC**

| **Variable** | | **Total population**  **N = 294** | **LSH**  **N = 43** | **LSCH+LSC**  **N = 208** | **TLH+LSC**  **N = 43** | **P*** |
| --- | --- | --- | --- | --- | --- | --- |
| BMI [Median (range)] | | 26.4 (17.7-37.2) | 25.2 (17.7-31.6) | 26.4 (19.2-37.2) | 28.2 (19.7-36.6) | 0.005^a^ |
| Age [Median (range)] | | 63.0 (28-84) | 49.0 (28-70) | 63.0 (37-84) | 66.0 (37-81) | <0.0001^a^ |
| Parity [Median (range)] | | 2.0 (0-9) | 2.0 (1-5) | 2.0 (0-9) | 2.0 (0-4) | 0.10^a^ |
| Cardiovascular disease [N (%)] | | 149 (50.7%) | 9 (20.9%) | 110 (52.9%) | 30 (69.8%) | <0.0001^b^ |
| Diabetes mellitus [N (%)] | | 40 (13.6%) | 1 (2.3%) | 30 (14.4%) | 9 (20.9%) | 0.03^b^ |
| Previous DVT or pulmonary embolism [N (%)] | | 39 (13.3%) | 3 (7.0%) | 30 (14.4%) | 6 (14.0%) | 0.42^b^ |
| Asthma [N (%)] | | 22 (7.5%) | 1 (2.3%) | 15 (7.2%) | 6 (14.0%) | 0.14^c^ |
| Previous abdominal surgical history [N (%)] | | 137 (46.6%) | 17 (39.5%) | 103 (49.5%) | 17 (39.5%) | 0.30^b^ |
| Previous gynecologic surgery [N (%)] | | 90 (30.6%) | 15 (34.9%) | 72 (34.6%) | 13 (30.2%) | 0.85^b^ |
| Previous POP surgery [N (%)] | | 6 (2.0%) | 2 (4.7%) | 4 (1.9%) | 0 (0.0%) | 0.40^c^ |
| Point C | POP Q stage | 19 (6.5%) | 5 (11.6%) | 13 (6.2%) | 1 (2.2%) | 0.002^c^ |
|  | POP Q stage II | 159 (54.1%) | 24 (55.8%) | 121 (58.2%) | 14 (32.6%) |  |
|  | POP Q stage III | 78 (26.5%) | 11 (25.6%) | 53 (25.5%) | 14 (32.6%) |  |
|  | POP Q stage IV | 38 (12.9%) | 3 (7.0%) | 21 (10.1%) | 14 (32.6%) |  |
| Point Ba | POP Q stage I | 3 (1.0%) | 1 (2.3%) | 2 (1.0%) | 0 (0.0%) | <0.0001^c^ |
|  | POP Q stage II | 65 (22.1%) | 28 (65.1%) | 32 (15.4%) | 5 (11.6%) |  |
|  | POP Q stage III | 165 (56.1%) | 11 (25.6%) | 141 (67.8%) | 13 (30.2%) |  |
|  | POP Q stage IV | 51 (17.4%) | 3 (7.0%) | 33 (15.8%) | 15 (34.9%) |  |
| Point Bp | POP Q stage I | 96 (32.7%) | 13 (30.2%) | 76 (36.5%) | 7 (16.3%) | <0.0001^c^ |
|  | POP Q stage II | 132 (44.9%) | 22 (51.2%) | 90 (43.3%) | 20 (46.5%) |  |
|  | POP Q stage III | 46 (15.6%) | 7 (16.3%) | 33 (15.8%) | 6 (13.9%) |  |
|  | POP Q stage IV | 20 (6.8%) | 1 (2.3%) | 9 (4.4%) | 10 (23.3%) |  |
| Stress urinary incontinence [N (%)] | | 87 (29.6%) | 11 (25.6%) | 61 (29.3%) | 15 (34.9%) | 0.63^b^ |
| Urge urinary incontinence [N (%)] | | 66 (22.4%) | 8 (18.6%) | 46 (22.1%) | 12 (279%) | 0.57^b^ |
| Hesitancy: a delay in initiating micturition [N (%)] | | 136 (46.3%) | 13 (30.2%) | 98 (47.1%) | 25 (58.1%) | 0.031^b^ |
| Urinary retention [N (%)] | | 126 (42.9%) | 21 (48.8%) | 93 (44.7%) | 22 (51.2%) | 0.69^b^ |
| Constipation [N (%)] | | 62 (21.1%) | 4 (9.3%) | 48 (23.1%) | 10 (23.3%) | 0.12^c^ |
| Anal incontinence [N (%)] | | 102/266 (38.3%) | 16/41 (39.0%) | 78/190 (41.1%) | 8/35 (22.9%) | 0.13^b^ |
| Pre-op UDI [median (range)] | | 51.2 (0-189) | 52.6 (5.8-164) | 54.1 (0-189) | 43.1 (2.5-183) | 0.46^a^ |
| Pre-op POPDI [median (range)] | | 68.5 (0-282) | 58.9 (10.7-152) | 72.6 (0-282) | 64.9 (376-192) | 0.30^a^ |
| Pre-op CRADI [median (range)] | | 35.1 (0-216) | 34.2 (0-164) | 37.1 (0-216) | 25.7 (0-113) | 0.51^a^ |
| Pre-op PFDI [median (range)] | | 171.7 (0-600) | 148.0 (16.5-442) | 178.4 (3.6-600) | 152.0 (9.6-418) | 0.35^a^ |

^a^ Kruskal-Wallis test; ^b^ Chi-square Test; ^c^ Fisher’s exact Test

BMI body mass index, DVT deep venouse thromboembolism
