# Supplementary material for: Pelvic organ prolapse and uterine preservation: a cohort study (POP-UP study)
Source: BMC Womens Health. 2021 Feb 17;21:72. doi: 10.1186/s12905-021-01208-5 (PMC7890869; doi:10.1186/s12905-021-01208-5)
Supplement: Supplementary file 2 — Additional file 2. Peri-Operative characteristics amongst women undergoing LSH, LSCH+LSC and TLH+LSC [file 12905_2021_1208_MOESM2_ESM.docx]

**Additional file 2: Peri-Operative characteristics amongst women undergoing LSH, LSCH+LSC and TLH+LSC**

| **Variable** | **Total population**  **N = 294** | **LSH**  **N = 43** | **LSCH+LSC**  **N = 208** | **TLH+LSC**  **N = 43** | **p** |
| --- | --- | --- | --- | --- | --- |
| Operating time [min] [Median (range)] | 120.5 (60-240) | 120.0 (70-225) | 120.5 (60-235) | 140.0 (60-240) | 0.049^a^ |
| Operating time more than 3 hours [N (%)] | 16 (5.4%) | 2 (4.7%) | 10 (4.8%) | 4 (9.3%) | 0.47^b^ |
| Blood loss [ml] [Median (range)] | 150 (50-1400) | 150 (50-1400) | 150 (50-800) | 250 (100-800) | <0.0001^a^ |
| Estimated blood loss more than 300 ml [N (%)] | 14 (4.7%) | 2 (4.7%) | 5 (2.4%) | 7 (16.3%) | 0.002^b^ |
| Perioperative blood transfusion | 2 (0.7%) | 1 (2.3%) | 1 (0.4%) | 0 (0.0%) | 0.50^b^ |
| Bladder injury [N (%)] | 10 (3.4%) | 2 (4.7%) | 5 (2.4%) | 3 (7.0%) | 0.19^b^ |
| Rectal injury [N (%)] | 0 (0.0%) | 0 (0.0%) | 0 (0.0%) | 0 (0.0%) | N/A |
| Vaginal injury [N (%)] | 2 (0.7%) | 0 (0.0%) | 1 (0.5%) | 1 (2.3%) | 0.50^b^ |
| Early postoperative complications Dindo-Clavien grade 0 [N (%)] | 281 (95.6%) | 41 (95.3%) | 199 (95,7%) | 41 (95.3%) | 0.81^b^ |
| Early postoperative complications Dindo-Clavien grade I [N (%)] | 6 (2.0%) | 1 (2.3%) | 4 (1.9%) | 1 (2.3%) |  |
| Early postoperative complications Dindo-Clavien grade II [N (%)] | 3 (1.0%) | 1 (2.3%) | 2 (1.0%) | 0 (0.0%) |  |
| Early postoperative complications Dindo-Clavien grade III [N (%)] | 4 (1.4%) | 0 (0.0%) | 3 (1.4%) | 1 (2.3%) |  |
| Prolonged hospitalization [N (%)] | 6 (2.0%) | 2 (4.7%) | 3 (1.4%) | 1 (2.3%) | 0.24^b^ |

^a^ Kruskal-Wallis Test; ^b^ Fisher’s Exact Test
