# Supplementary material for: Pelvic organ prolapse and uterine preservation: a cohort study (POP-UP study)
Source: BMC Womens Health. 2021 Feb 17;21:72. doi: 10.1186/s12905-021-01208-5 (PMC7890869; doi:10.1186/s12905-021-01208-5)
Supplement: Supplementary file 3 — Additional file 3. Post-operative follow-up at 3 months and at 12 months. [file 12905_2021_1208_MOESM3_ESM.docx]

**Additional file 3: Post-operative follow-up at 3 months and at 12 months.**

|  | **3 month follow-up**  **N=283** | | | | **12 month follow-up**  **N=271** | | | |
| --- | --- | --- | --- | --- | --- | --- | --- | --- |
|  | **Total**  **N = 283** | **Uterine sparing**  **(LSH)**  **N = 41** | **Concomitant hysterectomy**  **(LSCH+LSC & TLH+LSC)**  **N = 242** | **p** | **Total**  **N = 271** | **Uterine sparing**  **(LSH)**  **N = 38** | **Concomitant hysterectomy**  **(LSCH+LSC & TLH+LSC)**  **N = 233** | **p** |
| De novo SUI [N/N] (%) | 80/195 (41.0%) | 13/30 (43.3%) | 67/165 (40.6%) | 0.841 ^b^ | 43/185 (23.2%) | 12/28 (42.9%) | 31/157 (19.7%) | 0.014^b^ |
| De novo SUI [N/N] (%) ≥ weekly* | 63/195 (32.3%) | 10/30 (33.3%) | 53/165 (32.1%) | 1.000 ^b^ | 56/186 (30.1%)* | 11/29 (37.9%)* | 45/157 (28.7%) | 0.379^b^ |
| De novo UUI [N/N] (%) | 18/217(8.3%) | 1/33 (3.0%) | 17/184 (9.2%) | 0.321 ^b^ | 13/209 (6.2%) | 2/33 (6.1%) | 11/176 (6.3%) | 1.000^b^ |
| Improvement of UUI [N/N] (%) | 47/61 (77.0%) | 8/8 (100.0%) | 39/53 (73.6%) | 0.180 ^b^ | 51/59 (86.4%) | 6/6 (100.0%) | 45/53 (84.9%) | 0.348^b^ |
| Improvement of hesitancy: a delay in initiating micturition | 129/130 (99.2%) | 13/13 (100.0%) | 116/117 (99.1%) | 1.000 ^b^ | 125/126 (99.2%) | 13/13 (100.0%) | 111/113 (98.2%) | 1.000^b^ |
| De novo problem of hesitancy: a delay in initiating micturition [N/N] (%) | 2/151 (1.3%) | 1/28 (3.6%) | 1/123 (0.8%) | 0.337 ^b^ | 2/146 (1.4%) | 1/28 (3.6%) | 1/118 (0.8%) | 0.748^b^ |
| Improvement in urinary retention [N/N] (%) | 119/131 (90.8%) | 18/21 (85.7) | 102/110 (92.7%) | 0.382 ^b^ | 116/126 (92.1%) | 18/21 (85.7%) | 98/105 (93.3%) | 0.532^b^ |
| De novo urine retention [N/N] (%) | 5/149 (3.4%) | 1/20 (5.0%) | 4/129 (3.1%) | 0.519 ^b^ | 7/145 (4.8%) | 1/20 (5.0%) | 6/125 (4.8%) | 1.000^b^ |
| Improvement of AI [N/N] (%) | 67/87 (77.0%) | 12/13 (92.3%) | 55/74 (74.3%) | 0.283 ^b^ | 61/98 (62.2%) | 9/12 (75.0%) | 52/76 (68.4%) | 1.000^b^ |
| De novo AI [N/N] (%) | 24/161 (14.9%) | 5/26 (19.2%) | 19/135 (14.1%) | 0.548 ^b^ | 22/161 (13.7%) | 2/25 (8.0%) | 20/136 (14.7%) | 1.000^b^ |
| Improvement in constipation [N/N] (%) | 39/59 (66.1%) | 3/4 (75.0%) | 36/55 (65.5%) | 1.000 ^b^ | 44/60 (73.3%) | 3/4 (75.0%) | 41/56 (73.2%) | 1.000^b^ |
| De novo constipation [N/N] (%) | 15/221 (6.8%) | 3/36 (8.3%) | 12/185 (6.5%) | 0.717 ^b^ | 13/211 (6.2%) | 2/35 (5.7%) | 11/176 (6.3%) | 1.000^b^ |
| De novo diarrhoea [N/N] (%) | 1/283 (0.3%) | 0/41 (0.0%) | 1/242 (0.4%) | 1.000 ^b^ | 1/271 (0.4%) | 0/38 (0.0%) | 1/233 (0.4%) | 1.000^b^ |
| De novo painful defecation [N/N] (%) | 2/283 (0.7%) | 0/41 (0.0%) | 2/242 (0.8%) | 1.000 ^b^ | 3/271 (1.1%) | 0/38 (0.0%) | 3/233 (1.3%) | 1.000^b^ |
| Distance of the lowest anterior mesh extremity from the bladder neck <2.0 cm [N/N](%) | 260/274(94.9%) | 39/40(97.5%) | 221/234 (94.4%) | 0.701 ^b^ | 240/265(90.6%) | 36/39(92.3%) | 204/226 (90.3%) | 1.000 ^b^ |
| Regular shape of the mesh upon visualization of the whole mesh [N/N](%) | 244/266(91.7%) | 35/40(87.5%) | 209/226 (92.5%) | 0.345 ^b^ | 238/265(89.8%) | 32/39(82.1%) | 206/226 (91.2%) | 0.090 ^b^ |
| No folding of the mesh [N/N](%) | 248/268(92.5%) | 32/40(80.0%) | 216/228 (94.7%) | 0.004 ^b^ | 245/266(92.1%) | 32/39(82.1%) | 213/227 (93.8%) | 0.021 ^b^ |
| No mesh descent on Valsalva 196/226 (86.7%) [N/N](%) | 266/268(99.3%) | 39/40(97.5%) | 227/228 (99.6%) | 0.277 ^b^ | 252/254(99.2%) | 36/37(97.3%) | 216/217 (99.5%) | 0.271 ^b^ |
| Overall evaluation: all criteria for a properly placed mesh fulfilled [N/N](%) | 227/266(85.3%) | 31/40(77.5%) | 196/226 (86.7%) | 0.146 ^b^ | 214/254(84.3%) | 25/37(67.6%) | 189/217 (81.7%) | 0.006 ^b^ |

^a^ Mann-Whitney U test; ^b^ Fisher’s Exact Test; UUI: Urge urinary incontinence; SUI: Stress urinary incontinence.

* If TVT or bulking agens performed between 3 and 12 months, the woman remained in SUI group.
