# Supplementary material for: Pelvic organ prolapse and uterine preservation: a cohort study (POP-UP study)
Source: BMC Womens Health. 2021 Feb 17;21:72. doi: 10.1186/s12905-021-01208-5 (PMC7890869; doi:10.1186/s12905-021-01208-5)
Supplement: Supplementary file 4 — Additional file 4. Post hoc power calculation [file 12905_2021_1208_MOESM4_ESM.docx]

**Additional file 4: Post hoc power calculation**

Anterior compartment failure: 26/271 (9.6%) 8/38 (21.1%) 18/233 (7.7%)

The POWER Procedure

Pearson Chi-square Test for Two Proportions

Fixed Scenario Elements

Distribution Asymptotic normal

Method Normal approximation

Alpha 0.05

Group 1 Proportion 0.211

Group 2 Proportion 0.077

Group 1 Sample Size 38

Group 2 Sample Size 233

Number of Sides 2

Null Proportion Difference 0

Computed Power 0.686
